# Failure to replicate a superiority effect in crowding

**DOI:** 10.1038/s41467-025-56762-5

**Published:** 2025-02-14

**Authors:** Ayberk Ozkirli, David Pascucci, Michael H. Herzog

**Affiliations:** 1 Laboratory of Psychophysics, Brain Mind Institute, École Polytechnique Fédérale de Lausanne (EPFL), Lausanne, Switzerland; 2 Psychophysics and Neural Dynamics Lab, Lausanne University Hospital and University of Lausanne, Lausanne, Switzerland; 3 The Sense Innovation and Research Center, Lausanne, Switzerland

**Keywords:** Human behaviour, Object vision, Pattern vision

**Arising from** G. M. Cicchini et al. *Nature Communications* 10.1038/s41467-022-33508-1 (2022)

Cicchini et al.^1^ reported that performance in an orientation reproduction task can improve when a target is surrounded by flankers of similar orientation. They explained their results by a model, in which target and flanker orientations are optimally integrated. In their experiments, participants reproduced the orientation of a target (an oval shape), which was either elongated (easy task, reliable target) or rounded (harder task, unreliable target). The target was presented either alone (only the unreliable target) or with flankers of various orientations, which were also elongated or rounded oval shapes (see Fig. 1c, except for the top-right configuration). First, the authors observed that performance was enhanced in nearly all the flanked conditions compared to the single-target condition; i.e., they found a superiority effect. Second, performance followed a ‘U’-shaped pattern, with the largest improvements occurring when the target and flankers had identical orientations (Fig. 1b). These findings led the authors to conclude that “crowding improves overall performance”. Such *a superiority effect* contradicts typical findings in crowding research, where flankers can only deteriorate performance. Here, we failed to replicate their results. As usually observed in crowding, flankers deteriorated performance. We highlight some additional issues with their reasoning.

**Fig. 1:**
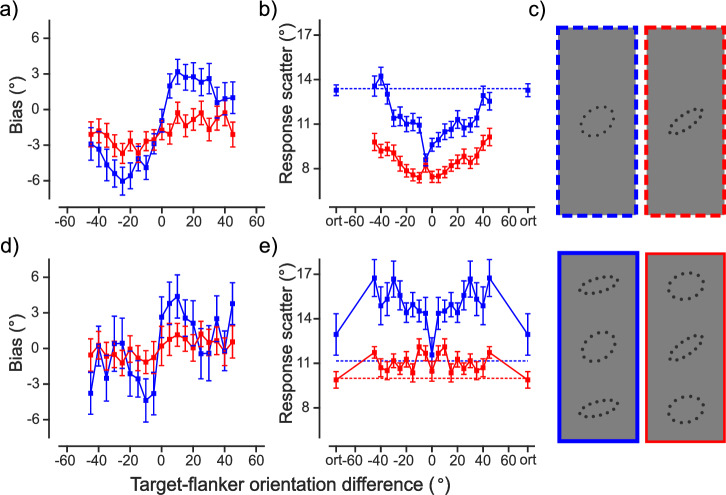
No superiority effect in crowding. **a**, **b** Results from Cicchini et al.^1^ with adjustments in the figure layout and without model fits (derived from two separate experiments: *N* = 8 for ‘ort’ and dashed baseline, and *N* = 10 for the remaining conditions), **d**, **e** our replication data (from one experiment: *N* = 20 participants), **c** Our flanked conditions at the bottom row and corresponding controls at the top (Cicchini et al.^1^ did not test the reliable single-target condition). **a**, **d** Mean response bias as a function of target-flanker orientation difference. The bias reflects the deviation of the reported target orientation towards the flankers’ orientation. **b**, **e** Mean response scatter (standard deviation) as a function of target-flanker orientation difference. The response scatter is the main variable of interest in our replication, showing how imprecise responses are, i.e., the response scatter reflects performance, which in most crowding studies is expressed as the percentage of correct responses or thresholds. Low response scatter values mean good performance. Dashed lines show the baseline for the reliable (red line) and unreliable (blue line) targets tested without flankers. Error bars in **a**, **b**, **d**, **e** represent SEM across participants. In Cicchini et al.^1^ the response scatter was largest in the baseline and orthogonal conditions (‘ort’ where flankers were oriented 90° away from the target), and decreased as the target-flanker orientation difference decreased, leading to a ‘U’-shaped function (see **b**). In addition, performance with flankers was almost always better than that with only the target, indicating a superiority effect. In our data, performance in the flanked conditions was almost always inferior to that in the corresponding single-target condition, particularly when the target-flanker orientation difference was between 5–45°, resulting in an M-shaped curve (see **e**) rather than a U-shaped one (see **b**).

We ran the same paradigm as in Cicchini et al.^1^ on a sample of 20 naive participants, doubling the sample size compared to the original study. The paradigm included the two flanked conditions (unreliable target with reliable flankers and reliable target with unreliable flankers), and the single-target condition for both reliable and unreliable targets (see Fig. 1c, and Section “Methods”). We found that performance with flankers was never superior to that in the corresponding single-target condition (see Fig. 1e). Moreover, performance exhibited a non-monotonic pattern: it was similar when the flankers were oriented 0° and 90° away from the target, with a clear deterioration in between, resulting in an ‘M’-shape d pattern rather than the ‘U’-shaped one  Cicchini et al.^1^ reported (see Fig. 1e). Although the M-shape was less pronounced when the target was reliable (red line in Fig. 1e) than when it was unreliable (blue line in Fig. 1e), statistical analyses led to the same conclusions for both conditions (see Table 1): crowding deteriorates, but does not improve performance.

**Table 1:** Two-sided paired t-tests for each condition compared to the corresponding baseline (single-target) condition

Target-flanker orientation difference	Target reliability	*p*	Cohen’s *d*	Bayes factor (BF_10_)	Posterior distribution	
					Median	95% CI
±5 to 45°	Unreliable	<0.001	1.457	6450.463	1.35	[0.725, 2.009]
	Reliable	<0.001	1.051	187.025	0.96	[0.421, 1.522]
0°	Unreliable*	0.771	0.068	0.247	0.058	[-0.357, 0.478]
	Reliable	0.532	0.142	0.279	0.123	[-0.284, 0.538]
90°	Unreliable**	0.182	0.328	0.555	0.282	[-0.154, 0.738]
	Reliable	0.869	-0.037	0.235	−0.032	[-0.441, 0.374]

Positive Cohen’s *d* indicates a larger response scatter in the flanked condition. The Bayes Factor (BF_10_) indicates evidence against the null hypothesis in favor of the alternative hypothesis. In this context, a BF_10_ > 100 provides decisive evidence against the null hypothesis, suggesting a performance deterioration with flankers. Conversely, a BF_10_ ∈ [0.1, 0.33) provides substantial evidence for the null hypothesis against the alternative, indicating no significant difference in performance due to flankers, while BF_10_ ∈ [0.33, 1) only provides anecdotal evidence for the null hypothesis. For the mid-range conditions (±5 to 45°), we averaged the response scatter across different target-flanker orientation differences before comparing them to the baseline. The last two columns summarize the posterior distributions, with median and 95% credible intervals, according to Bayesian paired t-test with Cauchy priors (default scaling factor *r* = 0.707). The results were robust regardless of the choice of prior and scaling factor. All the tests involved 20 participants except for conditions with asterisks. One participant was excluded due to large errors (>35°) in the flanked condition when the low-reliability target and high-reliability flankers had the same orientation (*), while two were excluded when they were oriented 90° away from each other (**).

The only instances where performance was comparable between flanked and unflanked conditions were when the target and flankers’ orientations were either identical (0°) or 90° apart (see Table 1 for Bayes factor analysis). Although small superiority or deterioration effects cannot be completely ruled out for these conditions due to limitations in statistical power (see Methods), our findings fail to replicate the superiority effects reported by the authors: with flankers, the response scatter was almost always larger than without, a pattern opposite to the one reported in Cicchini et al.^1^ (see Fig. 1b, e). Notably, the additional experiments included in their reply to this contribution also show that flankers almost always deteriorate performance (Cicchini et al., under review).

One possibility for the divergent results may be that Cicchini et al.^1^ had different participants in the main (*n* = 10, collection started in 2019) and control (*n* = 8, collected after revisions in 2022) conditions, which renders the data less comparable. Here, we measured all conditions within the same participants and in the same session, ensuring that crowding and control conditions could be compared using a repeated-measures design. Additionally, Cicchini et al.^1^ employed different target orientations in their flanked (35° or 55°) and single-target conditions (0°, 22.5°, 67.5° or 90° away from the vertical axis, see the published data in ref. ^2^). This could have led to performance discrepancies between the single-target condition and the flanked conditions, making direct comparisons less straightforward. Here, we ensured that the target orientations remained the same across both the flanked and single-target conditions (always 35° or 55° degrees away from the vertical axis).

Cicchini et al.’s^1^ findings are also in contradiction with previous studies. Livne and Sagi^3^, for instance, found that crowding in the fovea was strongest when the target and flankers’ orientations differed by approximately 45°, and was much reduced when they differed by 90° or when they shared the same orientation (resembling an M-shape, see Figure 4 in ref. ^3^), in line with our findings. Solomon et al.^4^ reported no improvement, but “losses of sensitivity” in most of the conditions with flankers, as compared to the single-target condition (with upside-down M-shapes as sensitivity is inversely related to response scatter, see Figure 4a in ref. ^4^). Surprisingly, Cicchini et al^1^, claim the opposite when referring to this study. Similarly, Glen and Dakin observed an M-shape when manipulating flanker positions horizontally^5^. The consistent observation of this shape across various studies, including our replication attempt, suggests that crowding cannot be explained by a simple cue integration model, as proposed by Cicchini et al^1^.

We agree on many of the theoretical points raised by Cicchini et al^1^., for instance, that there is no bottleneck and no irretrievable loss of information in crowding^6–8^, and that the global statistics of all the elements, rather than local interactions, are crucial^8–10^. Crowding may indeed be “a consequence of efficient exploitation of the spatial redundancies of the natural world”. However, this does not imply superiority effects. While crowding can be reduced by adding further flankers, i.e., uncrowding^6–8^, this never improves performance beyond the level in the single-target condition, i.e., a superiority effect. To the best of our knowledge, there are no superiority effects in crowding, especially not with such a large effect as reported in Cicchini et al.^1^. In general, superiority effects are relatively uncommon in vision research with simple displays.

In sum, we could not replicate the findings of a superiority effect in Cicchini et al.^1^, and therefore, we also found no evidence for optimal integration in crowding. Consistent with numerous previous studies, crowding only deteriorates performance.

## Methods

### Ethics statement

Data collection was done with the ethical approval of the local ethics committee (Commission cantonale d’éthique de la recherche sur l’être humain, protocol number: 2021-02270, title: Fundamental aspects of object recognition: general protocol) and in accordance with the Declaration of Helsinki. All participants provided informed consent and received monetary compensation for their participation.

### Apparatus

Stimuli were presented on a gamma-corrected LCD monitor (ASUS VG248QE, 24″, resolution: 1920 × 1080 pixels, refresh rate: 120 Hz, max. luminance set to 100 cd/m^2^) and were generated with an in-house toolbox written in MATLAB (R2013a) and the Psychophysics Toolbox, on a Windows-PC. Participants sat at approximately 57 cm from the computer screen, with the head held stable on a chin rest. The experiments were performed in a dimly lit room.

### Participants

Twenty naive, healthy participants (7 male, age range: 19–24) from the EPFL and the University of Lausanne took part in the study for monetary reward (25 CHF/h). This ensured sufficient statistical power (>0.8) to detect effect sizes above 0.66, as determined by Sensitivity Power Analysis^11^. All participants were naïve to the purpose of the experiments. Written informed consent was collected from all participants before the experiment.

### Stimuli and procedure

The parameters of the stimuli and paradigm closely followed the description in ref. ^1^. An example of the stimuli is provided in Fig. 1c. The target consisted of an oval shape (ellipse) created from 12 dark gray dots, each with a diameter of 0.3°. The oval target was presented in two conditions: alone or surrounded by two oval flankers. The center of the target was positioned 26° away from the fixation dot, whereas, when present, the flankers were positioned with their centers shifted 5.5° above and below the center of the target.

In accordance with the parameters used in the original work^1^, the ovals were either rounded (an aspect ratio of 1.4 with diameters 3.19° and 2.28°, low reliability) or elongated (an aspect ratio of 2.8 with diameters 3.48° and 1.23°, high reliability). Trials with low and high reliability of the target were pseudo-randomly intermixed within a block.

The target orientation was either 35° or 55° from the vertical axis. In the condition with flankers, the two flankers sharing the same orientation were yoked to the orientation of the target. As a result, flankers differed in orientation with respect to the target by either 90° or within a range of −45° to 45° in steps of 5°.

Unlike in ref. ^1^, where the target orientation in the condition without flankers could be 0°, 22.5°, 67.5°, or 90°, we used the same orientations for the target in all conditions (35° or 55°), even when presented without flankers (either clockwise or counterclockwise from vertical). Also, in contrast to the original study where the baseline (without flankers) and the 90° target-flanker orientation difference conditions were collected on a separate session following the reviewers’ request, each participant in our study was tested in all conditions within the same session. Trials with and without flankers were performed in separate blocks, with the order counter-balanced across participants. These procedures ensured a fully balanced, within-session repeated-measures design, with fully comparable target stimuli across all conditions.

Each trial started with a fixation dot presented for 500 ms on the left side of the screen. Participants were instructed to maintain fixation throughout the experiment. Then, the target with or without flankers was displayed for 167 ms. Following a stimulus-response interval of 500 ms, participants had to reproduce the perceived orientation of the target (referred to as ‘the ellipse’ in the unflanked conditions and as ‘the central ellipse’ in the flanked conditions) as quickly and accurately as possible by rotating a thin white response bar shown at fixation using the mouse. A new trial started after the response with a randomly sampled delay between 500-1000 ms in steps of 100 ms. The timing of events was kept consistent with the values reported in the original work, when available.

Each of the 20 participants completed 168 trials for each target reliability in both flanked and unflanked conditions, with a total of 13,440 trials (compared to 10 participants performing a total of 10,699 trials in ref. ^1^).

### Data analysis

Data cleaning and analysis also closely followed the procedure adopted in ref. ^1^. Trials with reaction times faster than 500 ms or slower than 3000 ms were removed, along with those featuring absolute adjustment errors larger than 35° (17% of the trials removed in total).

In contrast to ref. ^1^, we estimated the response bias and scatter separately for each participant, whereas in ref. ^1^ bias and scatter were estimated from the aggregated data. With this approach, we aimed to avoid the pitfall of aggregated group data^12–14^. The response bias was estimated as the average angular difference between the reported and the true target orientation, computed as a function of the target-flankers orientation difference. Positive values indicate that responses were biased towards the orientation of the flankers. The response scatter (standard deviation of adjustment errors) was estimated as a function of the target-flankers orientation difference in the conditions with flankers, and overall in the condition without flankers. Before the estimation of the response scatter, we first centered errors around 0° for each target orientation.

To increase statistical power in the estimated average response bias and scatter, we initially collapsed the data for negative and positive target-flanker orientation differences. This procedure involved ‘folding,’ which consisted of inverting the sign of adjustment errors for negative target-flanker orientation differences, and then evaluating the response bias and scatter as functions of the absolute target-flanker orientation difference, effectively doubling the number of data points for each orientation difference.

For graphical purposes, the plots in Fig. 1d, e depicts the patterns of response bias and scatter after folding, along with symmetric replicas for negative target-flanker orientation differences.

Statistical comparisons were performed using two-tailed paired *t*-tests between the main conditions of interest (see Table 1), with Cohen’s d serving as the estimate of the effect size. We also provide the Jeffreys, Zellner and Siow (JZS) Bayes Factor (BF_10_), showing the evidence for the alternative hypothesis over the null hypothesis. BFs were calculated using a Cauchy prior (with the default scaling factor, *r* = 0.707). Additionally, we report summaries of the posterior distributions for each comparison, using the central tendency (median) and 95% credibility interval (CI), which were estimated via Bayesian paired t-tests in JASP software. Further analyses confirmed that the results were robust against the use of different priors and scaling factors.

### Reporting summary

Further information on research design is available in the Nature Portfolio Reporting Summary linked to this article.

## Supplementary information

Reporting Summary

## Source data

Source Data

## Data Availability

The data used in this study is publicly available via Zenodo database^15^ in “.csv” format: 10.5281/zenodo.14617828. Source data are provided with this paper.

## Code availability

Analysis code used in this study is publicly available on CodeOcean^16^: https://codeocean.com/capsule/6180994/tree/v3/code/analysis_matters_arising.m.
