# Supplementary material for: Failure to replicate a superiority effect in crowding
Source: Nat Commun. 2025 Feb 14;16:1637. doi: 10.1038/s41467-025-56762-5 (PMC11829015; doi:10.1038/s41467-025-56762-5)
Supplement: Supplementary file 1 — Reporting Summary [file 41467_2025_56762_MOESM1_ESM.pdf]

nature portfolio

Corresponding author(s): Ayberk Ozkirli

Last updated by author(s): Jan 23, 2025

Reporting Summary

Nature Portfolio wishes to improve the reproducibility of the work that we publish. This form provides structure for consistency and transparency in reporting. For further information on Nature Portfolio policies, see our Editorial Policies and the Editorial Policy Checklist.

Statistics

For all statistical analyses, confirm that the following items are present in the figure legend, table legend, main text, or Methods section.

n/a	Confirmed	
☐	☒	The exact sample size (n) for each experimental group/condition, given as a discrete number and unit of measurement
☐	☒	A statement on whether measurements were taken from distinct samples or whether the same sample was measured repeatedly
☐	☒	The statistical test(s) used AND whether they are one- or two-sided<br>*Only common tests should be described solely by name; describe more complex techniques in the Methods section.*
☐	☒	A description of all covariates tested
☒	☐	A description of any assumptions or corrections, such as tests of normality and adjustment for multiple comparisons
☐	☒	A full description of the statistical parameters including central tendency (e.g. means) or other basic estimates (e.g. regression coefficient) AND variation (e.g. standard deviation) or associated estimates of uncertainty (e.g. confidence intervals)
☐	☒	For null hypothesis testing, the test statistic (e.g. F, t, r) with confidence intervals, effect sizes, degrees of freedom and P value noted<br>*Give P values as exact values whenever suitable.*
☐	☒	For Bayesian analysis, information on the choice of priors and Markov chain Monte Carlo settings
☒	☐	For hierarchical and complex designs, identification of the appropriate level for tests and full reporting of outcomes
☐	☒	Estimates of effect sizes (e.g. Cohen's d, Pearson's r), indicating how they were calculated

Our web collection on statistics for biologists contains articles on many of the points above.

Software and code

Policy information about availability of computer code

Data collection	Data collection Data was collected using an in-house toolbox employing Psychtoolbox 3.0.17.581582724 on MATLAB 2013a.
Data analysis	Data analysis Data was analyzed using MATLAB 2019b. The analysis code can be found on CodeOcean: https://doi.org/10.24433/CO.8256696.v3

For manuscripts utilizing custom algorithms or software that are central to the research but not yet described in published literature, software must be made available to editors and reviewers. We strongly encourage code deposition in a community repository (e.g. GitHub). See the Nature Portfolio guidelines for submitting code & software for further information.

Data

Policy information about availability of data

All manuscripts must include a data availability statement. This statement should provide the following information, where applicable:

- Accession codes, unique identifiers, or web links for publicly available datasets
- A description of any restrictions on data availability
- For clinical datasets or third party data, please ensure that the statement adheres to our policy

The data used in this study is publicly available via Zenodo database15 in ".csv" format: https://doi.org/10.5281/zenodo.14617828.

Research involving human participants, their data, or biological material

Policy information about studies with human participants or human data. See also policy information about sex, gender (identity/presentation), and sexual orientation and race, ethnicity and racism.

Reporting on sex and gender	7 male and 13 female participants took part in the study. Sex or gender was not considered for the experimental design and there were no sex-related analyses as it was not relevant for the research question.
Reporting on race, ethnicity, or other socially relevant groupings	Not applicable. We did not collect such data.
Population characteristics	People from university campuses in Lausanne (EPFL and UNIL). Ages between 19-24
Recruitment	The participants were recruited through an online platform, and random sampling was used.
Ethics oversight	Commission cantonale d'éthique de la recherche sur l'être humain (protocol number 2021-02270)

Note that full information on the approval of the study protocol must also be provided in the manuscript.

Field-specific reporting

Please select the one below that is the best fit for your research. If you are not sure, read the appropriate sections before making your selection.

☐ Life sciences ☒ Behavioural & social sciences ☐ Ecological, evolutionary & environmental sciences

For a reference copy of the document with all sections, see nature.com/documents/nr-reporting-summary-flat.pdf

Behavioural & social sciences study design

All studies must disclose on these points even when the disclosure is negative.

Study description	A replication study based on published paper about crowding (Cicchini et al., 2022). The participants were asked to reproduce the orientation of a target on each trial. The target was either presented alone or with flankers. The same participants completed trials from both conditions (repeated-measures design). Participants were blind to the aim of the experiment.
Research sample	People from university campuses in Lausanne (EPFL and UNIL). Ages between 19-24
Sampling strategy	Random sampling was used and participants were recruited through an online platform. N=20 participants ensured sufficient statistical power (>.8) to detect effect sizes above 0.66, as determined by Sensitivity Power Analysis.
Data collection	Target reliability conditions were intermixed across trials (flanked and unflanked in separate blocks). Naïve participants, dimly-lit room. The participants performed the practice trials when the researcher was in the room, however they were alone during the main blocks.
Timing	Data was collected in about 1.5 months, starting on 21.12.2022. A session lasted less than an hour.
Data exclusions	Exclusions were based on trials as in Cicchini et al. 2022. Reaction time below 500ms and above 3000ms, as well as large errors (above 35 degrees).
Non-participation	No participants dropped-out.
Randomization	All the experimental conditions were pseudo-randomized using interleaved design for both flanked and unflanked conditions. Every participant completed trials from both conditions.

Reporting for specific materials, systems and methods

We require information from authors about some types of materials, experimental systems and methods used in many studies. Here, indicate whether each material, system or method listed is relevant to your study. If you are not sure if a list item applies to your research, read the appropriate section before selecting a response.

Materials & experimental systems

n/a	Involved in the study
☒	☐ Antibodies
☒	☐ Eukaryotic cell lines
☒	☐ Palaeontology and archaeology
☒	☐ Animals and other organisms
☒	☐ Clinical data
☒	☐ Dual use research of concern
☒	☐ Plants

Methods

n/a	Involved in the study
☒	☐ ChIP-seq
☒	☐ Flow cytometry
☒	☐ MRI-based neuroimaging

Plants

Seed stocks	NA
Novel plant genotypes	NA
Authentication	NA
